# Unexpected episodes of cyanosis in late preterm and term neonates prompted admission to a neonatal care unit

**DOI:** 10.1186/s13052-017-0349-9

**Published:** 2017-04-14

**Authors:** Carlo Dani, Livia Drovandi, Giovanna Bertini, Chiara Poggi, Simone Pratesi

**Affiliations:** 1 Department of Neurosciences, Psychology, Drug Research and Child Health, Careggi University Hospital of Florence, Florence, Italy; 2 Division of Neonatology, Careggi University Hospital, Florence, Italy; 3 Division of Neonatology Careggi University Hospital, University of Florence, Largo Brambilla 3, 50141 Florence, Italy

**Keywords:** Cyanosis, Infant, Rooming-in

## Abstract

**Background:**

We studied late preterm and term infants who were admitted to our neonatal care unit in a tertiary hospital for unexpected episodes of cyanosis that occurred during rooming-in for evaluation of their frequency, most frequent associated diseases, and documentation of the diagnostic clinical approach.

**Methods:**

We carried out a retrospective study of infants with a gestational age ≥35 weeks who were admitted from the nursery with the diagnosis of cyanosis from January 2009 to December 2016. Exclusion criteria were the occurrence of acrocyanosis and the diagnosis of sudden unexpected postnatal collapse (SUPC).

**Results:**

We studied 49 infants with a mean gestational age of 38 ± 2 weeks. The frequency of admission for cyanosis was 1.8/1000 live births and was similar (*p* = 0.167) in late preterm and term infants. The majority of episodes occurred during the first 24 h of life (57%). Only 16 infants (33%) were discharged with a diagnosis, that was mostly (*n* = 5;10%) gastro-esophageal reflux.

**Conclusions:**

Unexpected episodes of cyanosis caused admission of 1.8/1000 live births to the neonatal care unit without differences between late preterm and term infants. These episodes occurred mainly during the first day of life and infants were mostly discharged without a known diagnosis.

## Background

The word cyanosis indicates the dark bluish discoloration of the skin, mucus membranes, and nail beds that becomes detectable when the levels of deoxygenated hemoglobin (Hb) in arterial blood exceed 3-5 g/dl [1]. It is sometimes difficult to detect cyanosis in the newborn due to factors such as skin colour, exposure to light, or presence of jaundice [2]. Since the relationship between cyanosis and pulse oximetry (SpO_2_) is directly related to the hemoglobin concentration, the percent desaturation required to produce the same grade of cyanosis is higher in anemic than in polycythemic infant [2].

Cyanosis can appear early after birth or later as an acute episode, often during crying or feeding when tissue oxygenation can decrease further [2]. The detection of cyanosis in infants is very important because it generally suggests inadequate peripheral tissue oxygenation and is one of the most accurate clinical signs of severe illness in infants during the first months of life [3].

Although cyanosis, hypoxemia, and hypoxia are not synonymous, they are interrelated and in clinical practice cyanosis can disclose the occurrence of tissue hypoxia whose pathogenesis is frequently due to cardiovascular and respiratory diseases or to less frequent pathologies such as hemoglobinopathies [4].

Therefore, the sudden appearance of an episode of cyanosis in a newborn in the hospital nursery who is expected to be healthy generally prompts his/her admission to neonatal care unit to monitor vital signs and carry out biochemical and instrumental diagnostic tests. Although cyanosis episodes are not rare in the neonatal period, there are few studies in the literature reporting on the epidemiology of this condition and on the diagnostic and therapeutic management of these patients [5].

Thus, the purpose of this study was to collect data on all late preterm and term infants who were admitted to our neonatal care unit in a tertiary hospital for unexpected episodes of cyanosis that occurred during rooming-in with mothers to evaluate their frequency and the most frequent pathologies, and document the performed diagnostic clinical approach.

## Methods

We carried out this retrospective study at the neonatal care unit of the Careggi University Hospital of Florence after the approval of the Tuscany pediatric ethics committee. Inborn infants with gestational age ≥35 weeks who were admitted from the nursery with the clinical diagnosis of cyanosis from January 2009 to December 2016 were included in the study. Infants with gestational age of 34 weeks were not studied because in our center they are always admitted to the neonatal care unit. Cyanosis was defined as the occurrence of dark blue discoloration of cutaneous and mucosal surfaces throughout the body (central cyanosis). Exclusion criteria were the occurrence of cyanosis limited to the extremities and lips (acrocyanosis) [6], and the diagnosis of sudden unexpected postnatal collapse (SUPC) as defined by the British Association of Perinatal Medicine: SUPC is (1) a sudden and unexpected postnatal collapse occurring within the first postnatal week (2) in an infant born >35 weeks gestational age, with a 5-min Apgar of >7, appearing well at birth and considered healthy, (3) requiring cardiopulmonary resuscitation and intensive care (with mechanical ventilation), and resulting in death or encephalopathy [7].

All infants were in complete rooming-in care where the mother and the baby stay together in the same room after birth for the duration of hospitalization [8, 9]. Thus, episodes of cyanosis might be detected by parents or caregivers.

Infants’ records were examined and the following data were recorded for each patient: gestational age, birth weight, gender, type of delivery, Apgar score at 1 and 5 min, patients’ age at episode of cyanosis, mode of recovery (spontaneous or stimulation), recurrent episodes, association with other signs/symptoms (i.e.: hypothermia, cardiac murmur, arrhythmias, hypotony, seizures, regurgitation/vomitus), blood tests [i.e.: blood gas analysis, cell counts, reactive C protein (RCP), C-troponin], cultures (blood and liquor), instrumental tests [chest X-rays, electrocardiogram (ECG), electroencephalogram (EEG), heart and/or cerebral ultrasound, cerebral nuclear magnetic resonance (NMR)], diagnosis at discharge, treatments, and admission duration.

Sepsis was diagnosed when patients developed clinical signs and symptoms associated with a positive blood and/or liquor culture. Suspected sepsis was diagnosed when patients developed clinical signs and symptoms with abnormal C-reactive protein without the confirmation of positive blood and/or liquor cultures.

### Statistical analysis

Patients’ clinical characteristics and recorded data were described as mean and standard deviation, median and range, or rate and percentage. Possible differences in the occurrence of cyanosis in subgroups of patients were evaluated with the *χ* ^2^ test for categorical variables. A *p* <0.05 was considered statistically significant.

## Results

During the study period 49 infants were eligible for the study and their characteristics are detailed in Table 1. One patients was excluded for SUPC. The frequency of admission for cyanosis was 1.8/1000 live births. The mean gestational age was 38 ±2 weeks, ranging from 35 to 41 weeks with 43 patients (88%) being term infants and 6 (12%) preterm. During the study period 1428 preterm infants and 25294 term infants (*n* = 26722) were assisted in complete rooming-in with mothers. Thus, the occurrence of cyanosis episodes was higher in preterm than in term infants (4.2/1000 vs. 1.7/1000 live births) but the difference was not significant (*p* = 0.167). The mean birth weight was 3240 ± 470 g; 2 of our patients were low birth weight infants (<2500 g) and 2 were macrosomic (>4000 g). Only 17 infants were males (35%).

**Table 1:** Clinical characteristics of infants. Mean ± (SD), rate and (%), or median and (range)

Clinical characteristics	*n* = 49
Gestational age (wks)	38 ±2
Birth weight (g)	3240 ± 470
Male gender	17 (35)
Cesarean section	14 (29)
Apgar score:	
-at 1 min	9 (8-10)
-at 5 min	10 (8-10)
Age at episode of cyanosis (h)	24 (3-120)
Mode of recovery:	
-spontaneous	40 (82)
-stimulation	9 (18)
Recurrent episodes	7 (14)
Associated signs/symptoms:	
-hypothermia	2 (4)
-cardiac murmur	3 (6)
-arrhythmias	2 (4)
-hypotony	9 (18)
-seizures	3 (6)
-regurgitation/vomitus	14 (29)
Hospital stay (d)	24 (3-44)
Maternal pregnancy disorders	
-gestational diabetes	10 (20)
-hypertensive disorders	5 (10)

Cyanosis occurred in infants at a median age of 24 (3-120) hours of life, with the majority of episodes occurring during the first 24 h of life (*n* = 28; 57%), and the remaining occurring at 25-48 h of life (n = 10; 20%), or after 48 h of life (*n* = 11; 23%). The lowest value of SpO_2_ in infants (*n* = 7; 14%) who had recurrent episodes of cyanosis was 83%.

Twenty-nine infants (59%) exhibited signs/symptoms associated with cyanosis and 4 (8%) had more than one. There was a maternal pregnancy disorder in 15 cases (31%) (Table 1).

It was possible to diagnose 16 (33%) infants (Table 2) and, interestingly, this occurred more frequently (71%) when they had recurrent episodes of cyanosis (14%). Appropriate therapies followed each diagnosis (unreported data).

**Table 2:** Diagnosis identified in our population. Rate and (%)

Diagnosis	*n* = 49
Gastro-esophageal reflux	5 (10)
Suspected sepsis	3 (6)
Inter-atrial septal defect	2 (4)
Genetic syndrome	2 (4)
Intraventricular hemorrhage	1 (2)
Seizures	1 (2)
Monolateral choanal atresia	1 (2)
Transient central apnea	1 (2)

All patients were given diagnostic tests and between laboratory tests blood gas analysis (100%) and cell counts (92%) were most commonly performed, while between instrumental tests heart (80%) and cerebral (88%) ultrasounds were the most common (Table 3).

**Table 3:** Diagnostic tests performed in our population. Rate and (%)

Test	*n* = 49
Laboratory and microbiological tests	
Gas analysis	49 (100)
Cell count	40 (92)
Reactive C Protein (RCP)	37 (76)
Procalcitonin (PCT)	29 (59)
Blood culture	9 (18)
Liquor culture	3 (6)
Instrumental tests	
Chest X-rays	27 (55)
Electrocardiogram (ECG),	28 (57)
Electroencephalogram (EEG),	15 (31)
Heart ultrasound	39 (80)
Cerebral ultrasound	43 (88)
Cerebral nuclear magnetic resonance (NMR)	4 (8)

## Discussion

In this study we report the frequency of unexpected episodes of cyanosis that occurred in our hospital in late preterm and term infants who were in complete rooming-in with mothers and that caused their admission to the neonatal care unit. We found a frequency of 1.8/1000 live births admitted that is lower than that previously reported by Casanueva et al. who found an admission rate of 5.6/1000 live births [5]. This difference might be because in this study infants who were born at 34 weeks of gestation were also included; moreover, the Casanueva’s definition of cyanosis was not well detailed and might have been different from ours [5].

It is interesting that the occurrence of cyanosis in our population was not significantly affected by the infants’ prematurity, since those born at 35-36 weeks had a similar frequency as term infants. It can be questioned whether our population size might affect this finding, however sample size (*n* = 26722) is reassuring from this point of view. This is very important because it is well known that late preterm infants commonly have a higher rate of medical complications (i.e.: respiratory distress, hypoglycemia, hyperbilirubinemia, etc.), prolonged hospital stay and need more medical resources when compared with matched full-term infants [10]. Nevertheless, the practice of assisting late preterm infants in complete rooming-in care is very diffused and our data seem to be reassuring regarding the safety of this procedure.

Most of patients in our population were female (65%), but this finding is not confirmed by a previous paper in which 51% of patients were female [5] and is probably due to the paucity of our sample.

We found that episodes of cyanosis frequently (67%) developed during the first day of life and we can speculate that this happens because this is the most critical period for the cardiovascular, respiratory, and metabolic transition from fetal to neonatal life [11].

The majority of infants exhibited signs/symptoms associated with cyanosis that sometimes were diagnosis-related (i.e.: cardiac murmur and inter-atrial defect, seizures and intraventricular hemorrhage), but sometimes were not (i.e.: regurgitation/vomitus). Therefore, unfortunately, it seems that the presence of these associations has limited usefulness in deciding or excluding the need for an infants’ admission for further evaluations.

It was possible to perform an exact diagnosis and provide proper treatment in a minority (33%) of infants in our population, in agreement with Casanueva’ study in which only 21% of patients were found to be affected by a specific pathology [5]. These results suggest that unexpected episodes of cyanosis of unknown etiology are often due to self-limiting transient functional events that do not require specific treatments. This speculation seems to be confirmed by the fact that when cyanosis recurs it is more probable (71%) to detect a pathology.

The most frequent diagnosis in our study was gastro-esophageal reflux and was based on clinical criteria (an episode of regurgitation/vomitus caused the admission for cyanosis and was followed by further episodes, etc.) and on recovery or improvement with thickening of breast milk/formula feedings [12] and/or H^2^-antagonists (i.e.: ranitidine) and/or alginate preparation treatment [13]. This finding partially agrees with a previous study [5] that found a gastro-esophageal reflux in a percentage of infants similar to ours (10 vs. 14%). On the contrary, we found that seizures were a rare cause of cyanosis (2%) which was the most frequent diagnosis (33.3%) in the Casanueva’ study [5].

Our patients were checked with biochemical, cultural, and instrumental tests to detect any possible infectious, metabolic, respiratory, cardiac, or neurological diseases as appropriate for their clinical condition, confirming that the development of an unexpected episode of cyanosis is resource-consuming [5] although efforts to ascertain a diagnosis are unsuccessful in the majority of cases.

We wonder if the crises of cyanosis of unknown origin that we observed in our patients might actually represent episodes of SUPC [7, 14] that were precociously and casually detected before the development of more severe symptoms requiring need of resuscitation, or if they represent a low risk condition that may be referred to as a brief resolved unexplained event (BRUE) requiring simple interventions such as positional changes, brief stimulation, or procedures to resolve airway obstruction [15]. We are not able to answer this question but we can report that, to the best of our knowledge, none of our patients developed subsequent episodes of cyanosis or more severe life-threatening events after their discharge from hospital. However, the issue of neonate’ surveillance during the first days of life is urgent and requires special and close attention to improve their safety while they are rooming in.

## Conclusions

We found 1.8/1000 live births unexpected episodes of cyanosis in infants without differences between late preterm and full-term who were in complete rooming-in with mothers which prompted their admission to neonatal care unit. We found that the majority of episodes of cyanosis occurred during the first day of life and that the majority of these infants were discharged without a known diagnosis.
